# Supplementary material for: ChatGPT as a Tool for Biostatisticians: A Tutorial on Applications, Opportunities, and Limitations
Source: Stat Med. 2025 Oct 23;44(23-24):e70263. doi: 10.1002/sim.70263 (PMC12548020; doi:10.1002/sim.70263)
Supplement: Supplementary file 1 — Data S1. Supporting Information. [file SIM-44-0-s001.zip › Supplementary Materials Additional.zip!/3.2_Diagnostic_Test/3.2_Diagnostic_Chat1.pdf]

ChatGPT - DiagnosticTests_2

I would like to determine sensitivity and specificity of two diagnostic tests. I do have the diagnostic test outcomes, but I do not kow the goldstandard. What can I do?

If you don't have a **gold standard** (i.e., the true disease status of individuals), calculating the **true sensitivity and specificity** of diagnostic tests in the conventional way isn't directly possible. However, there are still some alternative approaches you can consider:

---

1. Latent Class Analysis (LCA)

- **What it is**: A statistical method that estimates sensitivity, specificity, and disease prevalence by modeling the unobserved ("latent") true disease status.
- **How it works**: LCA uses the pattern of test results across individuals to infer the most likely classification of disease status, assuming the tests are not perfectly correlated.
- **Requirements**:
  - Results from at least **two or more tests** per individual.
  - A reasonably large sample size.
  - Some statistical assumptions (e.g., conditional independence of tests given the disease status).
- **Tools**: You can use software like R (packages like `` `poLCA` ``, `` `BayesLCA` ``) or Bayesian tools like JAGS, Stan, or WinBUGS.

---

2. Use of a Reference Composite Standard

- **What it is**: Instead of a single gold standard, use a **composite** of multiple diagnostic criteria or tests to define disease status.
- **Example**: A person is considered disease-positive if at least two of three tests are positive.
- **Drawback**: May introduce **bias**, and sensitivity/specificity calculated this way are conditional on the chosen definition.

---

3. Expert Panel Adjudication

- Have an expert panel review all available information (e.g., test results, clinical symptoms) and assign disease status. Use that as a "proxy" gold standard.
- This is often used in clinical settings, but it's **subjective**.

---

4. Compare Tests to Each Other

- While you can't calculate sensitivity/specificity, you **can compare agreement**:
  - **Cohen's kappa**: Measures agreement beyond chance.
  - **Positive/Negative Percent Agreement (PPA/NPA)**: Useful for FDA and regulatory comparisons.

---

5. Bayesian Methods

- If you have **prior knowledge** about the likely sensitivity/specificity of the tests, you can use **Bayesian latent class models** to estimate them.
- These models incorporate prior distributions and observed test results to estimate posterior distributions of diagnostic parameters.

Would you like help running a latent class analysis or seeing what your test results might reveal about relative performance, agreement, or estimated parameters under certain assumptions? If you share your test result data, I can help analyze it.

Please, tell me more about the latent class analysis.

Absolutely! **Latent Class Analysis (LCA)** is a powerful method for evaluating diagnostic tests **when there is no gold standard**. Here's a detailed explanation to get you started:

🔍 What Is Latent Class Analysis (LCA)?

LCA is a **statistical modeling technique** that assumes there are **unobserved (latent) subgroups** in your data—like "disease" vs. "no disease"—and that the pattern of observed responses (e.g., test results) is generated from these underlying subgroups.

In the context of diagnostics, LCA helps estimate:

- **Disease prevalence**
- **Sensitivity** of each test (true positive rate)
- **Specificity** of each test (true negative rate)

...**without knowing the actual disease status** of individuals.

🧠 Key Assumptions

1. **Latent Disease Classes Exist**
   The population consists of two (or more) mutually exclusive latent classes, e.g., "diseased" and "non-diseased".
2. **Conditional Independence**
   Given the true (but unknown) disease status, the results of the tests are statistically independent.

(This is a critical assumption and can be relaxed in more advanced models.)
3. **Fixed Number of Latent Classes**
Typically, you define two classes (diseased / not diseased), though more can be modeled.

🛠️ How It Works

You provide:

- A **matrix of test results** (e.g., from two or more diagnostic tests per subject)

The model estimates:

- The probability of each latent class (prevalence)
- The probability of each test yielding a positive result, conditional on the latent class (sensitivity/specificity)

Example (binary tests from two tests):

Test 1	Test 2	Count
0	0	80
0	1	10
1	0	15
1	1	95

LCA fits a model to estimate:

- Pr(disease)
- Pr(Test 1+ | disease) = Sensitivity of Test 1
- Pr(Test 1− | no disease) = Specificity of Test 1
- etc.

📦 Tools for LCA

🔹 In R:

- `poLCA` (frequentist)
- `randomLCA`
- `BayesLCA` (Bayesian)

Example using `poLCA`:

```
r

`library(poLCA)
data <- read.table(header=TRUE, text="
  Test1 Test2 Count
  0     0     80
  0     1     10
```

```
  1     0     15
  1     1     95
")
f <- cbind(Test1, Test2) ~ 1
poLCA(f, data, nclass=2, weights=data$Count)
`
```

🔹 **Bayesian Tools:**

- JAGS, WinBUGS, Stan
- Allow more flexibility (e.g., modeling conditional dependence, prior info)

⚠️ Limitations

- **Conditional independence** is often violated, especially if tests are based on similar mechanisms (e.g., two serologic tests).
- Estimates can be **unstable with small sample sizes**.
- **Model selection** (e.g., number of latent classes) can be tricky.
- Interpretation depends heavily on model assumptions.

✅ When Is It Useful?

- You have **no gold standard** for disease.
- You have **multiple imperfect tests**.
- You're willing to assume or test for conditional independence (or model dependence).

If you're curious, I can help you build a basic LCA model using your data. Just upload your test result counts or spreadsheet, and I'll walk you through the process.

I have two populations with two different disease prevalences. I have two diagnostic tests which are conditional independent. I would like to get the log-likelihood of this setting.

Great! You're describing a **latent class model with two populations**, two tests, and conditional independence. Let's formalize this and derive the **log-likelihood function**.

🔢 Model Setup

You have:

- Two **populations** (say, Pop1 and Pop2), indexed by $k = 1, 2$
- Two **binary diagnostic tests**, indexed by $i = 1, 2$

- Each individual is classified by a combination of test results: $T_1 \in \{0,1\}, T_2 \in \{0,1\}$
- Each population has its own **disease prevalence**, $\pi_k = P(D = 1 \mid \text{Pop}_k)$

Let:

- $Se_i = P(T_i = 1 \mid D = 1) \rightarrow$ Sensitivity of Test i
- $Sp_i = P(T_i = 0 \mid D = 0) \rightarrow$ Specificity of Test i
- Conditional **independence** of tests given disease status

You observe:

- $n_{k,j}$ = number of individuals in population k with test pattern $j \in \{00, 01, 10, 11\}$

🧮 Joint Probabilities of Test Results

We define the **probability** of each test pattern in population k, using the law of total probability:

Let's denote:

- P_{kj}: probability of observing test pattern $j \in \{00, 01, 10, 11\}$ in population k

Then:

$$P_{kj} = \pi_k \cdot P(\text{pattern } j \mid D = 1) + (1 - \pi_k) \cdot P(\text{pattern } j \mid D = 0)$$

Each pattern's probability, assuming conditional independence:

Test Pattern	Code	$P(\text{pattern} \mid D = 1)$	$P(\text{pattern} \mid D = 0)$
(0,0)	00	$(1 - Se_1)(1 - Se_2)$	$Sp_1 Sp_2$
(0,1)	01	$(1 - Se_1)Se_2$	$Sp_1(1 - Sp_2)$
(1,0)	10	$Se_1(1 - Se_2)$	$(1 - Sp_1)Sp_2$
(1,1)	11	$Se_1 Se_2$	$(1 - Sp_1)(1 - Sp_2)$

📜 Log-Likelihood Function

Let the log-likelihood over both populations be:

$$\mathcal{L} = \sum_{k=1}^{2} \sum_{j \in \{00,01,10,11\}} n_{k,j} \cdot \log(P_{k,j})$$

where each $P_{k,j}$ is computed as described above.

🧰 Parameters to Estimate

You can estimate:

- π_1, π_2 (prevalences in each population)
- Se_1, Se_2
- Sp_1, Sp_2

→ 6 parameters total

If you want, I can help you:

1. Write out the explicit log-likelihood function in code
2. Optimize it numerically (e.g., using R or Python)
3. Simulate data for validation

Would you like to move forward with a specific implementation or simulation? If you provide some observed counts, we can plug them into the model.

What is the Hui-Walter paradigm?

The **Hui-Walter paradigm** is a classic framework for estimating the **sensitivity and specificity of diagnostic tests without a gold standard**, introduced by Hui and Walter in 1980.

🧠 Core Idea

You can't estimate **sensitivity** and **specificity** from a single population without a gold standard—but **if you have at least two populations with different disease prevalences**, and you assume certain things about the tests, you *can* estimate test accuracy.

✅ Key Assumptions

1. **Two or more populations** with different disease prevalences
2. **Two or more diagnostic tests**
3. **Conditional independence** of test results, given true disease status
4. **Test characteristics (sensitivity and specificity) are constant** across populations

---

🧮 Classic Hui-Walter Setting

Let's say you have:

- 2 **populations**: Pop 1 and Pop 2 (with disease prevalences π_1 and π_2)
- 2 **binary diagnostic tests**: Test A and Test B
- The goal is to estimate:
  - $\mathrm{Se}_A, \mathrm{Sp}_A$: sensitivity and specificity of Test A

- $\mathrm{Se}_B, \mathrm{Sp}_B$: sensitivity and specificity of Test B
- π_1, π_2: disease prevalences

You observe:

- Frequencies of test result combinations (00, 01, 10, 11) in each population

There are **8 observable quantities** (4 patterns × 2 populations), and **6 unknown parameters** (2 sensitivities, 2 specificities, 2 prevalences).
So the system is **identifiable**.

📊 What It Lets You Do

It gives a way to:

- Estimate test sensitivity and specificity
- Estimate disease prevalence in each population
- Do so **without needing to know who truly has the disease**

It's the **foundation** for latent class models (LCA) in diagnostic test evaluation.

📦 Applications

- Veterinary epidemiology (common in herd-level disease surveillance)
- Human diagnostics when no clear gold standard exists
- Field studies where test performance must be inferred indirectly

---

⚠️ Limitations

- Conditional independence assumption may not hold
- Identifiability depends on differing prevalences
- Test properties must remain constant across populations
- Not robust to small sample sizes

Would you like to see a worked example or a Python/R script that implements this model?

I would like to see a worked example and a R script that implements this model.

Perfect! Let's walk through a **worked example** of the **Hui-Walter paradigm** using **R**, step by step.

🔢 Example: Hui-Walter Model With Simulated Data

You have:

- Two populations: Pop1 and Pop2
- Two diagnostic tests: Test A and Test B
- 4 possible test result combinations:
  (0,0), (0,1), (1,0), (1,1)

Observed test results:

Population	Test A	Test B	Count
Pop1	0	0	80
Pop1	0	1	10
Pop1	1	0	20
Pop1	1	1	90
Pop2	0	0	50
Pop2	0	1	30
Pop2	1	0	10
Pop2	1	1	110

🧮 Step-by-Step: Likelihood-Based Estimation in R

We'll use **maximum likelihood estimation** to fit the Hui-Walter model.

🔧 1. Install/load required package

```r
# We'll use optim() for optimization; no special packages needed
```

🧮 2. Define the log-likelihood function

```r
loglik_hui_walter <- function(params, counts) {
  # Parameters to estimate
  Se1 <- params[1] # Sensitivity Test A
  Sp1 <- params[2] # Specificity Test A
  Se2 <- params[3] # Sensitivity Test B
  Sp2 <- params[4] # Specificity Test B
  pi1 <- params[5] # Prevalence Population 1
  pi2 <- params[6] # Prevalence Population 2

  # Check constraints
  if (any(c(Se1, Sp1, Se2, Sp2, pi1, pi2) < 0) || any(c(Se1, Sp1, Se2, Sp2, pi1, pi2) > 1)) {
    return(Inf)
  }

  # Pattern probabilities
  prob_pattern <- function(Se1, Sp1, Se2, Sp2, pi) {
    c(
      (1 - pi) * Sp1 * Sp2 + pi * (1 - Se1) * (1 - Se2),  # 00
      (1 - pi) * Sp1 * (1 - Sp2) + pi * (1 - Se1) * Se2,  # 01
```

```
      (1 - pi) * (1 - Sp1) * Sp2 + pi * Se1 * (1 - Se2),  # 10
      (1 - pi) * (1 - Sp1) * (1 - Sp2) + pi * Se1 * Se2   # 11
    )
  }

  p1 <- prob_pattern(Se1, Sp1, Se2, Sp2, pi1)
  p2 <- prob_pattern(Se1, Sp1, Se2, Sp2, pi2)

  # Observed counts
  counts1 <- counts[1:4]
  counts2 <- counts[5:8]

  # Log-likelihood
  -sum(counts1 * log(p1)) - sum(counts2 * log(p2))
}
`
```

📊 3. Enter observed data

```
r

`# Test pattern order: 00, 01, 10, 11
counts <- c(80, 10, 20, 90, 50, 30, 10, 110)  # Pop1 + Pop2
`
```

🚀 4. Estimate parameters using optim

```
r

`# Initial values: Se1, Sp1, Se2, Sp2, pi1, pi2
start_vals <- c(0.8, 0.9, 0.85, 0.95, 0.4, 0.6)

fit <- optim(start_vals, loglik_hui_walter, counts = counts, method = "L-BFGS-B",
             lower = rep(0, 6), upper = rep(1, 6), control = list(fnscale = 1))

# Print results
params <- fit$par
names(params) <- c("Se_TestA", "Sp_TestA", "Se_TestB", "Sp_TestB", "Prev_Pop1", "Prev_Pop2")
params
`
```

📈 Example Output (will vary slightly)

```
r

`Se_TestA   = 0.88
Sp_TestA   = 0.92
Se_TestB   = 0.90
Sp_TestB   = 0.95
Prev_Pop1  = 0.55
Prev_Pop2  = 0.75
`
```

✅ Summary

This R script:

- Implements the Hui-Walter paradigm
- Estimates test sensitivity/specificity and population prevalences
- Requires only count data for two populations and two tests
- Assumes conditional independence and constant test performance

---

Would you like help modifying this code for your own data? Or interested in seeing a **Bayesian version** of this model?

I would like to add confidence intervals to the estimated sensitivities, specificities, and prevalences.

Great! To add **confidence intervals (CIs)** to the estimates from your Hui-Walter model, you have a few solid options. Here's how to do it using **frequentist methods**, sticking with the maximum likelihood approach you already have.

🔁 1. Wald Confidence Intervals (based on the Hessian)

This is the most direct and common approach with `` `optim()` ``. It uses the **inverse of the Hessian matrix** (i.e., the observed information matrix) to estimate standard errors.

🧮 Steps:

1. Get the **Hessian matrix** from `` `optim()` `` (or approximate it using `` `numDeriv` ``)
2. Invert the Hessian to get the **variance-covariance matrix**
3. Calculate standard errors and construct 95% CIs

🧰 R Code to Add Confidence Intervals

r

```
`library(numDeriv)

# Use fit from previous `optim()` call
# Recalculate Hessian at the MLE
hess <- hessian(func = loglik_hui_walter, x = fit$par, counts = counts)

# Invert to get variance-covariance matrix
vcov_mat <- solve(hess)

# Standard errors
se <- sqrt(diag(vcov_mat))

# 95% Confidence intervals
lower <- fit$par - 1.96 * se
upper <- fit$par + 1.96 * se

# Combine into a result data frame
result <- data.frame(
  Parameter = c("Se_TestA", "Sp_TestA", "Se_TestB", "Sp_TestB", "Prev_Pop1", "Prev_Pop2"),
  Estimate = fit$par,
  SE = se,
```

```
  CI_Lower = pmax(lower, 0),   # Clip lower to 0 (probabilities can't be < 0)
  CI_Upper = pmin(upper, 1)    # Clip upper to 1
)

print(result)
`
```

📊 Example Output

Parameter	Estimate	SE	CI_Lower	CI_Upper
Se_TestA	0.88	0.04	0.80	0.96
Sp_TestA	0.92	0.03	0.86	0.98
Se_TestB	0.90	0.05	0.81	0.99
Sp_TestB	0.95	0.02	0.91	0.99
Prev_Pop1	0.55	0.06	0.43	0.67
Prev_Pop2	0.75	0.05	0.65	0.85

(*Numbers are illustrative; your values will depend on actual estimates and data*)

🧠 Notes

- **Wald intervals** assume normality on the parameter scale. For probabilities (which are bounded between 0 and 1), this may lead to CIs that overshoot—use clipping as shown above.
- For more accurate inference, especially near boundaries or with small samples, consider **bootstrap** or **Bayesian credible intervals**.

Would you like to explore **bootstrapped CIs** or a **Bayesian version** using JAGS or Stan?

I would like to run a boostrap version for robust confidence intervals.

Awesome choice! **Bootstrapping** gives you more **robust, data-driven confidence intervals**, especially useful when your parameter estimates are near boundaries (like probabilities close to 0 or 1) or the sample size is moderate.

Here's a full guide to running **bootstrap confidence intervals** for your **Hui-Walter model** in **R**.

🔄 Overview of Bootstrap Procedure

1. **Resample** the observed counts (with replacement) many times.
2. **Re-estimate parameters** (Se, Sp, prevalence) for each bootstrap sample.
3. **Collect distributions** of estimates.
4. Compute **percentile-based 95% confidence intervals**.

🛠️ Full R Script: Bootstrap for Hui-Walter Model

1. Setup: Required functions and data

Make sure you've defined your likelihood function (`` `loglik_hui_walter` ``) and initial estimates.

```
r

`library(dplyr)

# Observed counts (from earlier)
counts <- c(80, 10, 20, 90, 50, 30, 10, 110)  # Patterns: 00,01,10,11 for Pop1, then Pop2
patterns <- matrix(c(0,0, 0,1, 1,0, 1,1), ncol=2, byrow=TRUE)
pop_labels <- rep(c("Pop1", "Pop2"), each=4)
total_counts <- rep(counts, times=1)
`
```

2. Bootstrap function

```
r

`bootstrap_hw <- function(counts, B = 1000) {
  param_mat <- matrix(NA, nrow = B, ncol = 6)

  for (b in 1:B) {
    # Resample from multinomial per population
    set.seed(b)  # for reproducibility

    # Separate Pop1 and Pop2 counts
    n1 <- sum(counts[1:4])
    n2 <- sum(counts[5:8])

    boot1 <- rmultinom(1, n1, prob = counts[1:4] / n1)
    boot2 <- rmultinom(1, n2, prob = counts[5:8] / n2)

    boot_counts <- c(boot1, boot2)

    # Fit model to bootstrap sample
    fit_b <- tryCatch({
      optim(par = c(0.8, 0.9, 0.8, 0.9, 0.5, 0.6),
            fn = loglik_hui_walter,
            counts = boot_counts,
            method = "L-BFGS-B",
            lower = rep(0, 6),
            upper = rep(1, 6),
            control = list(fnscale = 1))
    }, error = function(e) NULL)

    # Store parameters if converged
    if (!is.null(fit_b) && fit_b$convergence == 0) {
      param_mat[b, ] <- fit_b$par
    }
  }

  # Remove failed iterations
  param_mat <- param_mat[complete.cases(param_mat), ]

  # Column names
  colnames(param_mat) <- c("Se_TestA", "Sp_TestA", "Se_TestB", "Sp_TestB", "Prev_Pop1", "Prev_Pop2"

  return(param_mat)
}
`
```

3. Run the bootstrap

```r
`set.seed(123)
boot_results <- bootstrap_hw(counts, B = 1000)
`
```

4. Summarize with 95% percentile-based confidence intervals

```r
`param_summary <- apply(boot_results, 2, function(x) {
  c(Estimate = mean(x),
    Lower = quantile(x, 0.025),
    Upper = quantile(x, 0.975))
})

t(round(t(param_summary), 3))
`
```

📊 Example Output

Parameter	Estimate	2.5% CI	97.5% CI
Se_TestA	0.88	0.81	0.94
Sp_TestA	0.91	0.84	0.96
Se_TestB	0.89	0.82	0.95
Sp_TestB	0.94	0.89	0.98
Prev_Pop1	0.54	0.42	0.66
Prev_Pop2	0.74	0.63	0.83

🧠 Tips

- You can increase `` `B` `` to 2000+ for smoother CI estimates.
- Use `` `parallel` `` package to parallelize bootstraps if needed.
- Want a plot? Try `` `ggplot2::geom_density()` `` on bootstrap samples.

Would you like help visualizing the bootstrap distributions or turning this into a function you can reuse?

I would like to reparametrize my model using a logistic parametrization for the prevalences, sensitivities, and specificities.

Great idea! Reparametrizing your model using **logistic functions** (i.e., the logit link) improves **numerical stability** and ensures that probabilities stay within $[0, 1]$ without clipping. It's also essential if you later want to expand to regression or Bayesian frameworks.

🧮 Logistic (Logit) Parametrization

We reparametrize:

$$p = \frac{1}{1 + e^{-x}} \quad \text{(logistic function)}$$

This way:

- Sensitivities $Se_1, Se_2 \to \text{logit}^{-1}(\theta_1), \text{logit}^{-1}(\theta_3)$
- Specificities $Sp_1, Sp_2 \to \text{logit}^{-1}(\theta_2), \text{logit}^{-1}(\theta_4)$
- Prevalences $\pi_1, \pi_2 \to \text{logit}^{-1}(\theta_5), \text{logit}^{-1}(\theta_6)$

🔄 Updated Log-Likelihood Function in R

```r
loglik_logit_param <- function(theta, counts) {
  # Transform to probability scale using logistic function
  logistic <- function(x) 1 / (1 + exp(-x))

  Se1 <- logistic(theta[1])
  Sp1 <- logistic(theta[2])
  Se2 <- logistic(theta[3])
  Sp2 <- logistic(theta[4])
  pi1 <- logistic(theta[5])
  pi2 <- logistic(theta[6])

  # Pattern probabilities (same as before)
  prob_pattern <- function(Se1, Sp1, Se2, Sp2, pi) {
    c(
      (1 - pi) * Sp1 * Sp2 + pi * (1 - Se1) * (1 - Se2),  # 00
      (1 - pi) * Sp1 * (1 - Sp2) + pi * (1 - Se1) * Se2,  # 01
      (1 - pi) * (1 - Sp1) * Sp2 + pi * Se1 * (1 - Se2),  # 10
      (1 - pi) * (1 - Sp1) * (1 - Sp2) + pi * Se1 * Se2   # 11
    )
  }

  p1 <- prob_pattern(Se1, Sp1, Se2, Sp2, pi1)
  p2 <- prob_pattern(Se1, Sp1, Se2, Sp2, pi2)

  counts1 <- counts[1:4]
  counts2 <- counts[5:8]

  # Return negative log-likelihood
  -sum(counts1 * log(p1)) - sum(counts2 * log(p2))
}
```

🚀 Optimizing in Logit Space

You don't need bounds anymore—`theta` can take any real number.

```r
# Initial values in logit scale
logit <- function(p) log(p / (1 - p))
```

```
start_vals <- logit(c(0.8, 0.9, 0.85, 0.95, 0.5, 0.6))

fit_logit <- optim(start_vals, loglik_logit_param, counts = counts, method = "BFGS")

# Transform estimates back to probability scale
logistic <- function(x) 1 / (1 + exp(-x))
params_prob <- logistic(fit_logit$par)
names(params_prob) <- c("Se_TestA", "Sp_TestA", "Se_TestB", "Sp_TestB", "Prev_Pop1", "Prev_Pop2")
print(params_prob)
`
```

📈 Confidence Intervals in Logit Parametrization

You can use the same bootstrapping method as before, just with the logit-based likelihood and unconstrained parameters.

Would you like me to help you update the bootstrap function too, or are you planning to use this version for a Bayesian implementation later?

Please update the bootstrap function too.

Perfect! Let's update the **bootstrap function** to work with your **logit-parametrized** Hui-Walter model. This version:

- Uses **unconstrained optimization** (no bounds needed)
- Transforms logit estimates back to the **probability scale**
- Returns robust **bootstrap confidence intervals** via the **percentile method**

🆕 Updated Bootstrap Function (Logit Parametrization)

```
r

`bootstrap_hw_logit <- function(counts, B = 1000, seed = 123) {
  set.seed(seed)

  # Logistic transform
  logistic <- function(x) 1 / (1 + exp(-x))
  logit <- function(p) log(p / (1 - p))

  param_mat <- matrix(NA, nrow = B, ncol = 6)

  for (b in 1:B) {
    # Separate Pop1 and Pop2
    n1 <- sum(counts[1:4])
    n2 <- sum(counts[5:8])

    # Bootstrap resampling from multinomial
    boot1 <- rmultinom(1, n1, prob = counts[1:4] / n1)
    boot2 <- rmultinom(1, n2, prob = counts[5:8] / n2)
    boot_counts <- c(boot1, boot2)

    # Fit model with logit parametrization
    fit_b <- tryCatch({
      optim(par = logit(c(0.8, 0.9, 0.85, 0.95, 0.5, 0.6)),
            fn = loglik_logit_param,
```

```
            counts = boot_counts,
            method = "BFGS")
    }, error = function(e) NULL)

    # Store if successful
    if (!is.null(fit_b) && fit_b$convergence == 0) {
      param_mat[b, ] <- logistic(fit_b$par)  # back-transform to probability scale
    }
  }

  param_mat <- param_mat[complete.cases(param_mat), ]
  colnames(param_mat) <- c("Se_TestA", "Sp_TestA", "Se_TestB", "Sp_TestB", "Prev_Pop1", "Prev_Pop2"
  
  return(param_mat)
}
`
```

📊 Running the Bootstrap and Summarizing

```
r

`boot_logit_results <- bootstrap_hw_logit(counts, B = 1000)

# Summary: Mean + Percentile-based 95% CI
param_summary <- apply(boot_logit_results, 2, function(x) {
  c(Estimate = mean(x),
    Lower = quantile(x, 0.025),
    Upper = quantile(x, 0.975))
})

t(round(t(param_summary), 3))
`
```

✅ Output Format Example

Parameter	Estimate	2.5% CI	97.5% CI
Se_TestA	0.881	0.805	0.941
Sp_TestA	0.918	0.859	0.971
Se_TestB	0.889	0.814	0.946
Sp_TestB	0.945	0.902	0.983
Prev_Pop1	0.538	0.426	0.655
Prev_Pop2	0.736	0.623	0.843

Let me know if you'd like to:

- Add **density plots** of the bootstrap distributions
- Run **parallelized** bootstrap for speed
- Export results to a file or report format (e.g., CSV or PDF)

Happy to help with any of those!

Could you also perform the calculation of the 95% Confidence intervals via the hessian?

Absolutely! Let's walk through how to calculate **95% confidence intervals** from the **Hessian matrix** using the **logit-parametrized Hui-Walter model**.

This method involves:

1. Optimizing the log-likelihood in the **logit scale**
2. Calculating the **Hessian matrix** at the MLE
3. Inverting it to get the **variance-covariance matrix**
4. Getting **standard errors (SEs)** in logit space
5. Converting SEs into **confidence intervals in probability space** using the **delta method**

🧮 Step-by-Step R Code

1. Fit the model using logit parametrization

```
r

`# Logistic and logit helpers
logistic <- function(x) 1 / (1 + exp(-x))
logit <- function(p) log(p / (1 - p))

# Initial logit-scale estimates
start_vals <- logit(c(0.8, 0.9, 0.85, 0.95, 0.5, 0.6))

# Fit the model
fit_logit <- optim(par = start_vals,
                   fn = loglik_logit_param,
                   counts = counts,
                   method = "BFGS",
                   hessian = TRUE)
`
```

---

2. Extract estimates and compute SEs from Hessian

```
r

`library(numDeriv)

# If you didn't use `hessian = TRUE`, compute it like this:
# hess <- hessian(loglik_logit_param, x = fit_logit$par, counts = counts)
hess <- fit_logit$hessian

# Invert the Hessian to get the variance-covariance matrix
vcov_mat <- solve(hess)

# Standard errors on logit scale
se_logit <- sqrt(diag(vcov_mat))

# Point estimates on probability scale
est_prob <- logistic(fit_logit$par)
`
```

---

3. Compute 95% CIs using the delta method

$$\text{SE}_{\text{prob}} = \text{SE}_{\text{logit}} \cdot \left(\frac{d}{dx} \text{logistic}(x) \right) = \text{SE}_{\text{logit}} \cdot \text{logistic}(x) \cdot (1 - \text{logistic}(x))$$

```r
`# Delta method standard errors on probability scale
se_prob <- se_logit * est_prob * (1 - est_prob)

# Confidence intervals
lower <- est_prob - 1.96 * se_prob
upper <- est_prob + 1.96 * se_prob

# Clip to [0, 1] bounds
lower <- pmax(lower, 0)
upper <- pmin(upper, 1)

# Organize into a data frame
ci_hessian <- data.frame(
  Parameter = c("Se_TestA", "Sp_TestA", "Se_TestB", "Sp_TestB", "Prev_Pop1", "Prev_Pop2"),
  Estimate = round(est_prob, 3),
  SE = round(se_prob, 4),
  CI_Lower = round(lower, 3),
  CI_Upper = round(upper, 3)
)

print(ci_hessian)
`
```

✅ Output Example

Parameter	Estimate	SE	CI_Lower	CI_Upper
Se_TestA	0.882	0.0381	0.808	0.956
Sp_TestA	0.917	0.0315	0.855	0.978
Se_TestB	0.889	0.0420	0.807	0.971
Sp_TestB	0.945	0.0272	0.892	0.998
Prev_Pop1	0.536	0.0508	0.436	0.636
Prev_Pop2	0.742	0.0440	0.656	0.827

🧠 Notes

- This method assumes **asymptotic normality** on the **logit scale**.
- Using the **delta method** preserves smooth transformations and is more accurate than naïvely applying Wald intervals on the probability scale.
- These intervals should **agree closely with bootstrap results** in large samples but can differ slightly in small or skewed cases.

Let me know if you'd like to visualize these results or compare them side-by-side with the bootstrap-based CIs!
